# HuR Regulates GATA3-Driven Type 2 Inflammation in CD4^+^ T cells and ILC2 in Airway Inflammation

**DOI:** 10.64898/2026.04.23.720195

**Published:** 2026-04-27

**Authors:** Fatemeh Fattahi, Laura Yaekle, Julia Holden, Brandon Tepper, Kareem Hussein, Joshua Meier, Liang Xu, Srilaxmi Nerella, Jing Lei, Kelley Bentley, Marc Hershenson, Steven Huang, Ulus Atasoy

**Affiliations:** 1 Division of Allergy and Clinical Immunology, Department of Internal Medicine, University of Michigan Medical School, Ann Arbor, MI; 2 University of Kansas Cancer Center, The University of Kansas Medical Center, Kansas City, KS; 3 Department of Medicine, Division of Pulmonary and Critical Care Medicine, UCSF, San Francisco, CA; 4 Departments of Pediatrics, Molecular and Integrative Physiology, University of Michigan, Ann Arbor, MI; 5 Division of Pulmonary and Critical Care Medicine, Department of Internal Medicine, University of Michigan Medical School, Ann Arbor, MI; 6 Section of Allergy-Immunology, Ann Arbor VA Health System, Ann Arbor, MI

## Abstract

Type 2–high asthma is driven by coordinated GATA3-dependent programs in CD4^+^ T cells and group 2 innate lymphoid cells (ILC2). Although biologics targeting IL-4, IL-5, or IL-13 benefit subsets of patients, many remain symptomatic, suggesting that upstream regulatory mechanisms may sustain type 2 inflammation. We investigated whether HuR (*ELAVL1*), an RNA-binding protein that stabilizes *GATA3* and Th2 cytokines mRNA, regulates type 2 inflammatory programs in allergic asthma. Using a house dust mite (HDM) model *in vivo*, HuR inhibition with the small molecule KH-3 reduced lung inflammation, suppressed Th2 cytokine expression, accelerated *Gata3* mRNA decay in lung CD4^+^ T cells, and attenuated airway hyperresponsiveness toward control levels. In *ex vivo*–activated human lung CD4^+^ T cells, KH-3 accelerated *GATA3* mRNA decay with minimal effects on *RORC* or *TBX21* and selectively reduced Th2 cytokine secretion, while IL-10 and IL-2 were unchanged. Similarly, ILC2s isolated from peripheral blood mononuclear cells (PBMCs) of type 2–high asthmatic donors showed reduced *GATA3* mRNA stability and diminished Th2 cytokine production following KH-3 treatment. Single-cell transcriptomic analysis of bronchoalveolar lavage fluid after allergen challenge demonstrated co-enrichment of *ELAVL1* and *GATA3* within Th2 clusters in human airways. Together, these findings identify HuR as a post-transcriptional regulator of GATA3-driven type 2 inflammation in allergic asthma.

## Graphical Abstract

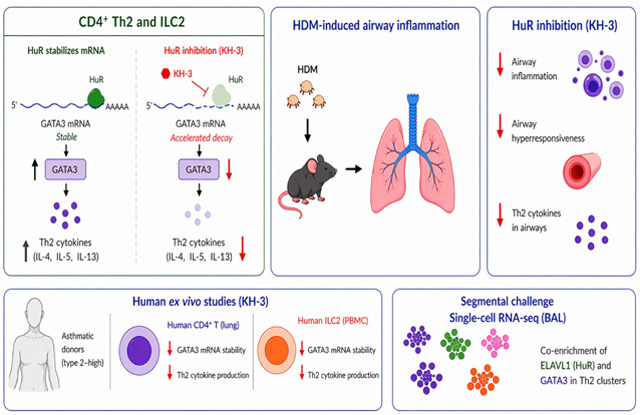

## Introduction

Allergic asthma remains a major cause of chronic respiratory morbidity worldwide, affecting more than 25 million individuals in the United States alone and contributing substantially to healthcare burden. A substantial proportion of patients exhibit a type 2–high inflammatory endotype, defined by eosinophilic airway inflammation, elevated IgE, and increased production of the canonical type 2 cytokines IL-4, IL-5, and IL-13 (1, 2). These cytokines orchestrate many of the pathologic features of asthma, including eosinophil recruitment and survival, goblet cell metaplasia, mucus hypersecretion, and airway hyperresponsiveness (3). Although inhaled corticosteroids and biologic therapies block IL-4, IL-5, IL-13 (4, 5), or upstream epithelial alarmins such as TSLP, IL-33, and IL-25 (6–8), have improved outcomes for many patients, a meaningful fraction remains symptomatic, underscoring the need for therapeutic strategies that target upstream or parallel pathways capable of broadly suppressing the inflammatory network driving disease.

Type 2 inflammation is orchestrated by coordinated transcriptional and post-transcriptional programs operating across both adaptive and innate lymphoid compartments (9, 10). GATA3 is the lineage-defining transcription factor for Th2 cells and group 2 innate lymphoid cells (ILC2), driving Th2 differentiation (IL-4/IL-5/IL-13) in CD4^+^ T cells and effector function in ILC2 responding to epithelial alarmins (IL-33/IL-25/TSLP) (11–17). Because GATA3 sits at the center of this program across multiple immune cell types (10), mechanisms that sustain its expression may amplify allergic inflammation; however, the processes that maintain GATA3 abundance under inflammatory conditions remain incompletely defined. Although GATA3 has been extensively studied as a transcriptional regulator (11), far less is known about its post-transcriptional control. RNA-binding proteins (RBPs) regulate mRNA stability, translation, and localization, thereby shaping both the magnitude and duration of immune responses (18–20). HuR (*ELAVL1*), which binds AU-rich elements (AREs) in the 3′ UTRs of numerous transcripts, is a ubiquitously expressed RBP that stabilizes inflammatory mRNAs and prolongs gene expression (21–24). The *GATA3* mRNA contains AREs within its 3′ UTR, making it a plausible HuR target (25, 26). Consistent with this, our prior work demonstrated that HuR directly binds and stabilizes *GATA3* transcripts in peripheral CD4^+^ T cells from human asthmatic donors, particularly in type 2–high disease, and that T cell–specific HuR ablation in mice significantly attenuates allergic airway inflammation *in vivo*, establishing a causal role for HuR in Th2-driven pathology (26).

Current cytokine-directed therapies act downstream of this broader inflammatory program (4). Because type 2 immunity depends on coordinated expression of lineage-defining and effector transcripts (IL-4/IL-5/IL-13), targeting a shared upstream regulator may provide broader and potentially more durable therapeutic benefit than blocking individual cytokines. KH-3, a small-molecule HuR inhibitor developed by the Xu lab, disrupts HuR–RNA interactions, reduces HuR-mediated transcript stabilization, and has shown *in vivo* activity with acceptable tolerability across diverse murine disease models, including cardiac hypertrophy, nephritis, pancreatic cancer cachexia, and pancreatic cancer metastasis (27–35). These properties support its use as a pharmacologic tool to interrogate HuR-dependent inflammatory pathways *in vivo*.

If HuR is required to maintain GATA3 expression across both adaptive (Th2) and innate (ILC2) compartments (9, 10, 25, 26), then pharmacologic inhibition with KH-3 would be expected to shorten *GATA3* mRNA half-life, suppress downstream type 2 cytokine production, and attenuate allergic airway inflammation. By targeting a shared post-transcriptional regulator, this approach may overcome a key limitation of existing biologic therapies that act on individual downstream mediators rather than the broader regulatory network.

Here, we tested this hypothesis using complementary murine and human systems, including an HDM-induced mouse model, primary murine and human lung CD4^+^ T cells, PBMC-derived ILC2 from asthmatic donors, and single-cell transcriptomic analysis of allergen-challenged human bronchoalveolar lavage fluid. This integrated, multi-level approach allowed us to determine whether the HuR–GATA3 axis is conserved across species and immune compartments and to evaluate whether pharmacologic inhibition of HuR represents a novel upstream therapeutic strategy for type 2–high asthma.

## Results

### HDM-induced type 2 airway inflammation and *Gata3* expression are attenuated by HuR inhibition *in vivo* (Figure 1)

**Figure 1.**
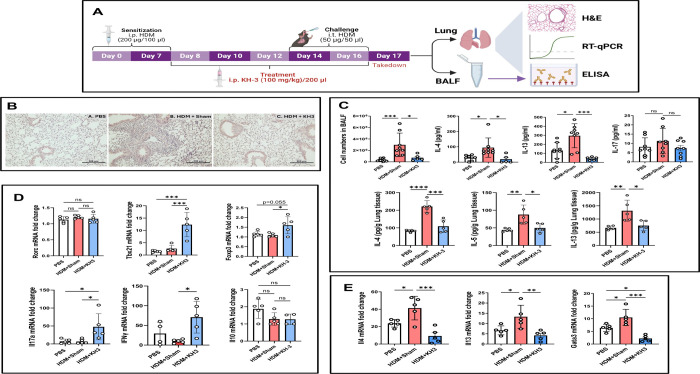
HDM-induced type 2 airway inflammation and *Gata3* expression are attenuated by HuR inhibition *in vivo.* **(A)** Experimental design of the HDM-induced allergic asthma model. Mice were sensitized intraperitoneally with HDM on days 0 and 7 and challenged intratracheally on days 14 and 16. KH-3 was administered intraperitoneally during the challenge phase. Lungs and bronchoalveolar lavage fluid (BALF) were harvested on day 17 for histological, molecular, and cellular analyses. **(B)** Representative hematoxylin and eosin (H&E)–stained lung sections from PBS−, HDM+sham−, and HDM+KH-3–treated mice. HDM+sham mice exhibited marked peribronchial and perivascular inflammatory infiltrates, which were substantially reduced following KH-3 treatment. **(C)** Th2 cytokine protein levels measured by ELISA in BALF (upper panels) and lung homogenates (lower panels). KH-3 significantly reduced IL-4, IL-5, and IL-13 compared with HDM+sham controls. **(D)** RT-qPCR analysis of lung homogenates showing reduced expression of *Gata3* and Th2 cytokines (*Il4*, *Il5*, *Il13*) following KH-3 treatment, with minimal effects on non–Th2 lineage markers. **(E)** BALF cellular analysis demonstrating reduced total leukocyte counts and decreased eosinophils, lymphocytes, and neutrophils in KH-3–treated mice compared with HDM+sham controls. Data are presented as mean ± SD from at least two independent experiments. Statistical analyses in panels (C), (D), and (E) were performed using one-way ANOVA. *p < 0.05; **p < 0.01; ***p < 0.001; ****p < 0.0001.

We next asked whether pharmacologic inhibition of HuR modulates established type 2 airway inflammation in an allergen-driven model (Figure 1A). Mice were sensitized and challenged with HDM, and KH-3 was administered during the challenge phase to target ongoing inflammation. At the study endpoint, bronchoalveolar lavage fluid (BALF) and lung tissues were collected for histologic, cytokine, and transcriptional analyses. Histologic evaluation confirmed robust allergic airway inflammation in HDM+sham mice, characterized by dense peribronchial and perivascular inflammatory infiltrates, whereas PBS-treated controls showed minimal inflammation. In contrast, KH-3–treated mice exhibited visibly reduced inflammatory cuffing and cellular infiltration around the airways and vessels (Figure 1B). Consistent with these findings, HDM challenge induced marked increases in Th2 cytokines. As shown in Figure 1C, in BALF, IL-4 and IL-13 were significantly elevated in HDM+sham mice compared with PBS controls and were reduced with KH-3 treatment (*p < 0.05–0.001 vs. HDM+sham). IL-17 levels, however, were not significantly altered across groups (Figure 1C). Similar trends were observed in lung homogenates, where IL-4, IL-5, and IL-13 were significantly increased following HDM exposure and reduced with KH-3 treatment (all *p < 0.05 vs. HDM+sham), although levels remained above baseline (Figure 1C). At the transcriptional level, RT-qPCR analysis of lung tissue showed that HDM exposure increased expression of Th2-associated genes, including *Gata3*, *Il4*, and *Il13* (Figure 1E). KH-3 treatment significantly reduced the expression of these transcripts (*p < 0.05–0.001 vs. HDM+sham). In contrast, expression of non–Th2 lineage markers (*Rorc*, *Tbx21*, and *Il10*) was not significantly altered, while *Foxp3* showed a modest trend toward increase that did not reach statistical significance (*p =* 0.055) (Figure 1D), suggesting selective modulation of the Th2 program.

Together, these data demonstrate that pharmacologic inhibition of HuR with KH-3 attenuates HDM-induced type 2 airway inflammation at histologic, molecular, and cellular levels *in vivo*.

### HuR inhibition accelerates *Gata3* mRNA decay and diminishes GATA3 protein in allergen-experienced lung CD4^+^ T cells (Figure 2)

**Figure 2.**
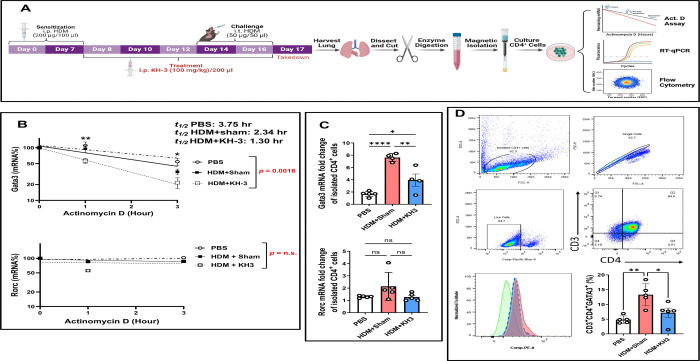
HuR inhibition accelerates *Gata3* mRNA decay and diminishes GATA3 protein in allergen-experienced lung CD4^+^ T cells. **(A)** Experimental schematic. Mice were sensitized and challenged with HDM and treated with KH-3 or vehicle. On day 17, lungs were harvested, enzymatically digested, and CD4^+^ T cells were isolated by magnetic column–based separation. Cells were acutely transferred to culture for actinomycin D (ActD) chase assays, RT-qPCR analysis, or flow cytometric evaluation. **(B)** ActD chase assays demonstrating mRNA stability in isolated lung CD4^+^ T cells. RNA was collected at 1 and 3 hours following ActD treatment. *Gata3* mRNA decay was significantly accelerated in HDM+KH-3–treated mice compared with HDM+sham controls (*t½* values: PBS, 3.75 hr; HDM+sham, 2.34 hr; HDM+KH-3, 1.30 hr; treatment effect *p =* 0.0016). In contrast, *Rorc* mRNA stability was not significantly altered (*p = ns*). Decay curves were analyzed by two-way ANOVA (n = 5 mice per group). **(C)** Steady-state mRNA expression of *Gata3* and *Rorc* in isolated lung CD4^+^ T cells. HDM challenge increased *Gata3* expression, which was significantly reduced by KH-3 treatment, whereas *Rorc* expression remained unchanged. Statistical comparisons were performed using one-way ANOVA (n = 5 per group). **(D)** Representative flow cytometry plots and quantification of CD3^+^CD4^+^GATA3^+^ cells showing reduced GATA3 protein expression in lung CD4^+^ T cells from HDM+KH-3–treated mice compared with HDM+sham controls. Statistical analysis was performed using one-way ANOVA. Data are presented as mean ± SD (n = 5 mice per group). *p < 0.05; **p < 0.01; ns, not significant.

Because *Gata3* is a central transcriptional driver of Th2 responses, we next asked whether KH-3 directly affects *Gata3* mRNA stability in lung CD4^+^ T cells recovered from allergen-challenged mice (Figure 2A). As outlined in Figure 2A, mice were subjected to HDM sensitization and challenge with concurrent KH-3 treatment, after which lung CD4^+^ T cells were isolated and analyzed *ex vivo* using ActD chase assays, RT-qPCR, and flow cytometry.

In PBS controls, *Gata3* mRNA decayed gradually following ActD treatment, whereas HDM challenge accelerated *Gata3* mRNA turnover (Figure 2B). Notably, KH-3 treatment further enhanced this decay, resulting in a significantly greater reduction in *Gata3* mRNA over time compared with HDM+sham controls (*p =* 0.0016, *t½* values: PBS, 3.75 hr; HDM+sham, 2.34 hr; HDM+KH-3, 1.30 hr). In contrast, *Rorc* mRNA stability was not significantly altered across PBS, HDM+sham, and HDM+KH-3 groups (*p = ns*), indicating that HuR inhibition selectively destabilizes *Gata3* transcripts rather than globally affecting mRNA turnover. Steady-state transcript analysis in purified lung CD4^+^ T cells paralleled these findings (Figure 2C). HDM exposure significantly increased *Gata3* mRNA expression compared with PBS controls, whereas KH-3 treatment reduced *Gata3* transcript levels relative to HDM+sham (*p < 0.05–0.0001). In contrast, *Rorc* expression remained unchanged across groups (*p = ns*), further supporting selective modulation of the Th2 transcriptional program.

At the protein level, flow cytometric analysis of lung CD3^+^CD4^+^ T cells demonstrated that HDM challenge increased the frequency of GATA3^+^ cells, whereas KH-3 treatment significantly reduced GATA3 expression (Figure 2D; *p < 0.05–0.01 vs. HDM+sham). Representative gating and histograms confirmed a shift toward lower GATA3 expression in KH-3–treated mice. Together, these data demonstrate that in allergen-experienced lung CD4^+^ T cells, pharmacologic inhibition of HuR with KH-3 accelerates *Gata3* mRNA decay and reduces GATA3 protein expression, supporting a model in which HuR maintains the Th2 transcriptional program by stabilizing *Gata3* transcripts.

### Allergen-induced airway hyperresponsiveness is improved by pharmacologic inhibition of HuR (Figure 3)

**Figure 3.**
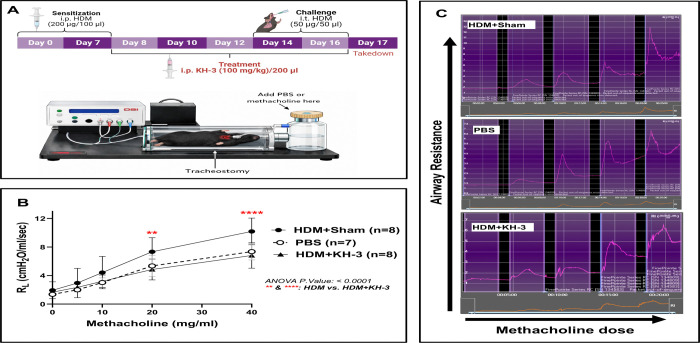
Allergen-induced airway hyperresponsiveness is improved by pharmacologic inhibition of HuR. **(A)** Experimental schematic and setup for measurement of airway resistance using invasive plethysmography following HDM sensitization/challenge and KH-3 treatment. **(B)** Airway resistance (R_L_) in response to increasing doses of methacholine. HDM+sham mice exhibited exaggerated airway hyperresponsiveness compared with PBS controls, which was significantly attenuated by KH-3 treatment (two-way ANOVA, overall *p* < 0.0001; post hoc comparisons between HDM+sham and HDM+KH-3 are indicated on the graph). Data represent combined results from two independent experiments (PBS, n = 7; HDM+sham, n = 8; HDM+KH-3, n = 8) and are presented as mean ± SD. **(C)** Representative airway resistance tracings from PBS, HDM+sham, and HDM+KH-3 groups across methacholine doses.

To determine whether modulation of HuR and *Gata3* translates into functional improvement, we assessed airway hyperresponsiveness in the HDM model using invasive plethysmography in intubated, tracheostomized mice (Figure 3A). HDM+sham mice exhibited the expected increase in airway responsiveness, with significantly higher airway resistance (R_L_) compared with PBS controls across increasing methacholine doses (Figure 3B). KH-3 treatment markedly attenuated this response, resulting in significantly lower airway resistance compared with HDM+sham mice, particularly at higher methacholine concentrations (two-way ANOVA, overall *p* < 0.0001; Figure 3B). Airway resistance in KH-3–treated mice approached levels observed in PBS controls. Representative airway resistance tracings further demonstrated reduced peak responses in KH-3–treated mice compared with HDM+sham animals (Figure 3C). These findings indicate that KH-3 treatment improves a key physiological feature of allergic asthma, consistent with its effects on airway inflammation and Th2 responses (Figures 1 and 2).

### HuR inhibition impairs Th2 effector responses in murine CD4^+^ T cells *in vitro* by (Figure 4)

**Figure 4.**
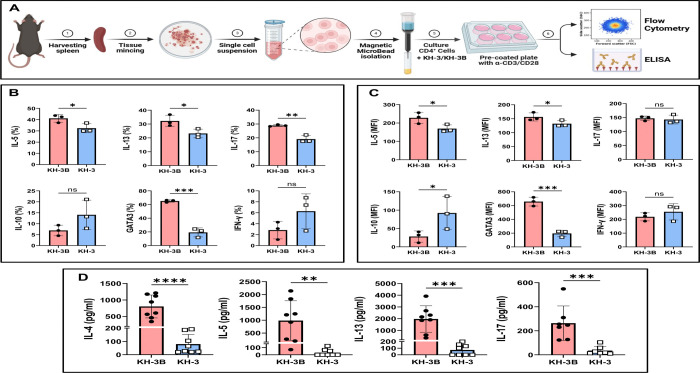
HuR inhibition impairs Th2 effector responses in murine CD4^+^ T cells *in vitro.* **(A)** Experimental workflow. Splenic CD4^+^ T cells were isolated by magnetic bead separation, pretreated with KH-3 or the inactive analogue KH-3B for 2 hours, and then activated with anti-CD3/CD28 for 4 days. Cells were subsequently analyzed by flow cytometry and ELISA. **(B)** Intracellular cytokine staining showing the percentage (%) of IL-5^+^, IL-13^+^, IL-17^+^, IL-10^+^, GATA3^+^, and IFN-γ^+^ CD4^+^ T cells. KH-3 treatment significantly reduced IL-5, IL-13, IL-17, and GATA3 expression compared with KH-3B controls, while IFN-γ and IL-10 were minimally affected. **(C)** Mean fluorescence intensity (MFI) analysis confirming selective suppression of Th2-associated markers following KH-3 treatment. **(D)** ELISA quantification of cytokines in culture supernatants showing reduced secretion of IL-4, IL-5, IL-13, and IL-17 following KH-3 treatment compared with KH-3B controls. Data are presented as mean ± SD from three independent experiments. Statistical comparisons were performed using two-tailed Student’s t test. *p < 0.05; **p < 0.01; ***p < 0.001; ****p < 0.0001; ns, not significant.

We next asked whether the effects of KH-3 on Th2 programming could be reproduced in a reductionist setting using isolated murine CD4^+^ T cells (Figure 4A). As outlined in Figure 4A, splenic CD4^+^ T cells were isolated, pretreated with KH-3 or the inactive analog KH-3B, and activated with anti-CD3/CD28, followed by assessment of intracellular cytokine production, transcription factor expression, and cytokine secretion. After 4 days of activation, intracellular staining revealed that KH-3 significantly reduced the proportion of IL-5^+^ and IL-13^+^ CD4^+^ T cells compared with KH-3B controls (*p < 0.05; Figure 4B). IL-17^+^ cells were also reduced (**p < 0.01), whereas IL-10^+^ and IFN-γ^+^ populations were not significantly altered (*p = ns*). Notably, KH-3 treatment markedly decreased the frequency of GATA3^+^ cells (***p < 0.001), indicating suppression of the Th2 transcriptional program.

Analysis of mean fluorescence intensity (MFI) supported these findings (Figure 4C). KH-3 treatment reduced IL-5 and IL-13 expression levels (*p < 0.05), and strongly decreased GATA3 MFI (***p < 0.001), while IL-17 and IFN-γ expression remained unchanged (*p = ns*). In contrast, IL-10 MFI was increased following KH-3 treatment (*p < 0.05), suggesting a potential shift toward a regulatory phenotype. Cytokine measurements in culture supernatants were consistent with the intracellular data (Figure 4D). KH-3 treatment significantly reduced secretion of IL-4 (****p < 0.0001), IL-5 (**p < 0.01), IL-13 (***p < 0.001), and IL-17 (***p < 0.001) compared with KH-3B controls. Together, these data demonstrate that, in the absence of other cell types, pharmacologic inhibition of HuR directly suppresses Th2 effector function in murine CD4^+^ T cells, accompanied by reduced GATA3 expression and cytokine production. These findings are consistent with the *in vivo* data (Figures 1–3) and support a model in which HuR acts cell-intrinsically to sustain *Gata3*-dependent Th2 responses.

### HuR inhibition selectively attenuates type 2 cytokine production in *ex vivo*–activated human lung CD4^+^ T cells (Figure 5)

**Figure 5.**
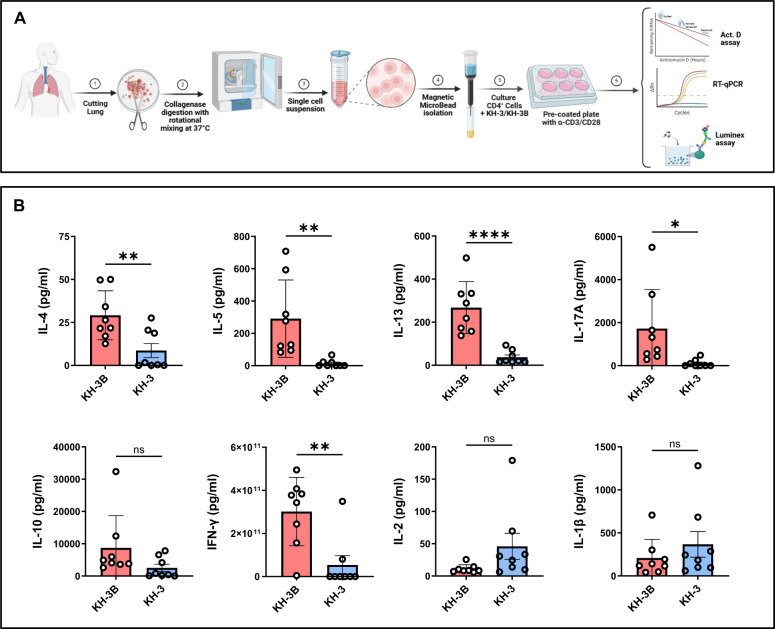
HuR inhibition selectively attenuates type 2 cytokine production in *ex vivo*–activated human lung CD4^+^ T cells. **(A)** Human lung CD4^+^ T cells were isolated from healthy control donors by magnetic bead separation following lung tissue mincing and collagenase digestion. Cells were pretreated with KH-3 or the inactive analogue KH-3B (5 μM) for 2 hours; cells were then activated with anti-CD3/CD28 for 4 days. Culture supernatants were collected and analyzed using a Luminex multiplex assay. Cells were processed for downstream molecular analyses as described in Figure 6. **(B)** KH-3 significantly reduced secretion of IL-4, IL-5, IL-13, IL-17A, and IFN-γ compared with KH-3B controls, whereas IL-10, IL-2, and IL-1β were not significantly changed (as indicated). Data are presented as mean ± SD. Each donor contributed samples from two lung regions (4 total biological samples), and each sample was split into KH-3B vs KH-3 conditions, yielding 8 data points per group. Statistical analyses were performed using paired two-tailed Student’s t test. *p < 0.05; **p < 0.01; ****p < 0.0001; ns, not significant.

To assess whether the HuR–GATA3 axis identified in mouse studies is conserved in human airways, we analyzed CD4^+^ T cells isolated from human lung tissue obtained from healthy donors (Figure 5A). As outlined in Figure 5A, lung tissue was enzymatically digested to generate single-cell suspensions, followed by magnetic isolation of CD4^+^ T cells. Cells were treated with KH-3 or the inactive analog KH-3B and activated prior to analysis of cytokine production. Using a Luminex platform, we found that KH-3 significantly reduced secretion of key Th2 cytokines, including IL-4 (**p < 0.01), IL-5 (**p < 0.01), and IL-13 (****p < 0.0001), compared with KH-3B-treated controls (Figure 5B). KH-3 also reduced IL-17A (*p < 0.05) and IFN-γ (**p < 0.01), although the magnitude of suppression was most pronounced for Th2-associated cytokines. In contrast, levels of IL-10, IL-2, and IL-1β were not significantly altered by KH-3 treatment (all *p* = *ns*), indicating that HuR inhibition does not broadly suppress cytokine production but preferentially targets Th2 responses.

Because each donor contributed paired KH-3B and KH-3 samples, these findings reflect within-donor comparisons and are not driven by inter-individual variability. Together, the data demonstrate that pharmacologic inhibition of HuR selectively attenuates type 2 cytokine production in human lung CD4^+^ T cells, supporting conservation of this pathway across species and motivating further mechanistic analysis of GATA3 regulation.

### HuR blockade destabilizes *GATA3* mRNA and reduces GATA3 expression in *ex vivo*–activated human lung CD4^+^ T cells (Figure 6)

**Figure 6.**
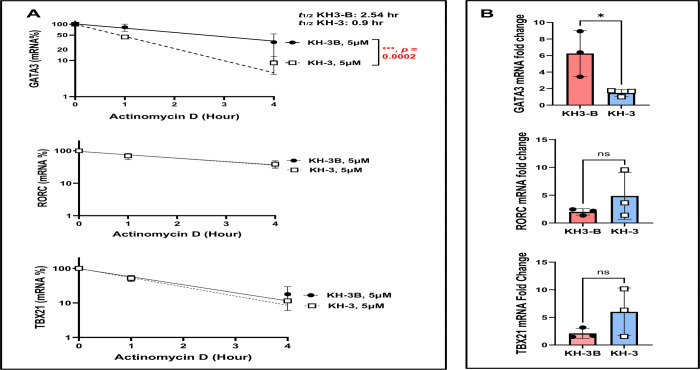
HuR blockade destabilizes *GATA3* mRNA and reduces GATA3 expression in *ex vivo*–activated human lung CD4^+^ T cells. **(A)** Actinomycin D (ActD) chase assays, performed following the experimental design described in Figure 5A, demonstrate accelerated decay of *GATA3* mRNA in KH-3–treated cells compared with KH-3B controls (two-way ANOVA; treatment effect *p =* 0.0002). The calculated mRNA half-life (*t½*) of *GATA3* was 2.54 hours in KH-3B–treated cells and 0.09 hours in KH-3–treated cells, whereas *RORC* and *TBX21* were not significantly altered. **(B)** Steady-state mRNA levels of *GATA3*, *RORC*, and *TBX21* measured by RT-qPCR in activated CD4^+^ T cells after 4 days of stimulation; statistical comparisons were performed using two-tailed Student’s t test. Data are presented as mean ± SD from independent human lung donors. *p < 0.05; ns, not significant.

To define the mechanism underlying reduced type 2 cytokine production (Figure 5), we next examined whether KH-3 affects *GATA3* mRNA stability in human lung CD4^+^ T cells (Figure 6A). As shown in Figure 6A, CD4^+^ T cells isolated from lung tissue of healthy human donors (n= 4) were pretreated with KH-3 or KH-3B, activated with anti-CD3/CD28, and subjected to ActD chase assays to assess mRNA decay.

*GATA3* mRNA decayed more rapidly in KH-3–treated cells compared with KH-3B controls, with a significantly steeper decline over time. The calculated mRNA half-life (t½) of *GATA3* was 2.54 hours in KH-3B–treated cells and 0.09 hours in KH-3–treated cells (*p =* 0.0002; Figure 6A). In contrast, the decay kinetics of *RORC* and *TBX21* were not significantly altered by KH-3 treatment (both *p = ns*), indicating selective destabilization of *GATA3* transcripts.

Consistent with these findings, steady-state RT-qPCR analysis demonstrated that KH-3 significantly reduced *GATA3* mRNA levels in activated human lung CD4^+^ T cells compared with KH-3B controls (*p < 0.05; Figure 6B). In contrast, expression of *RORC* and *TBX21* remained unchanged (*p = ns*), further supporting selective targeting of the GATA3 axis.

Together, these data demonstrate that pharmacologic inhibition of HuR with KH-3 destabilizes *GATA3* mRNA and reduces its steady-state expression in human lung CD4^+^ T cells, without broadly affecting other lineage-defining transcription factors. These findings mirror the selective effects observed in murine CD4^+^ T cells (Figure 2) and provide a mechanistic basis for reduced type 2 cytokine production in *ex vivo*–activated human airway T cells from healthy donors.

### Type 2 cytokine secretion by human peripheral ILC2s is reduced by HuR inhibition (Figure 7)

**Figure 7.**
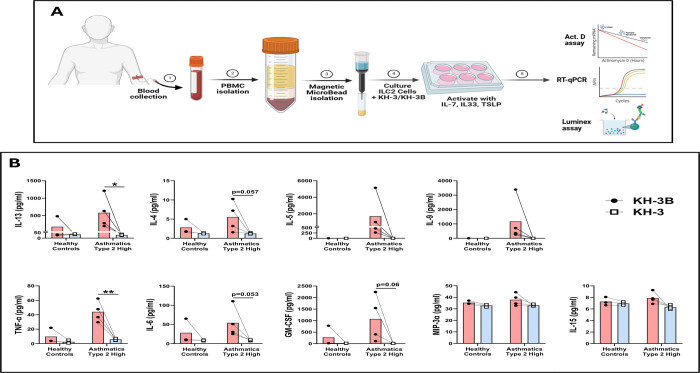
Type 2 cytokine secretion by human peripheral ILC2s is reduced by HuR inhibition. **(A)** Human PBMC-derived ILC2 were isolated and treated with KH-3 or the inactive analogue KH-3B, then activated with IL-7, IL-33, and TSLP prior to supernatant collection for Luminex analysis and cells were analyzed for molecular assay as will described in Figure 8. **(B)** KH-3 reduced cytokine production in type 2–high asthmatic donors, including IL-13, IL-4, IL-5, IL-9, TNF-α, IL-6, and GM-CSF, with minimal effects on MIP-3α and IL-15. Responses are shown for healthy controls (n = 3) and type 2–high asthmatic (n = 4) donors, with paired lines indicating matched conditions within each donor. Statistical comparisons were performed using paired two-tailed Student’s t test; exact p values are shown on the graphs where indicated. Data are presented as mean ± SD. *p < 0.05; **p < 0.01; ns, not significant.

Given the growing recognition that ILC2s contribute to persistent and steroid-refractory type 2 inflammation, we next tested whether HuR inhibition affects ILC2 effector responses (Figure 7). PBMC-derived ILC2s from healthy controls and type 2–high asthmatic donors were isolated, treated with KH-3 or the inactive analog KH-3B, and stimulated with IL-7, IL-33, and TSLP, as outlined in Figure 7A. In cells from asthmatic donors, KH-3 significantly reduced secretion of TNF-α (**p < 0.01) and IL-13 (*p < 0.05) (Figure 7B). IL-4, IL-6, and GM-CSF showed a trend toward reduction, with p values indicated in Figure 7B, whereas IL-5, IL-9, MIP-3α, and IL-15 were not significantly altered (*p = ns*). Paired analyses within each donor demonstrated a consistent downward shift in canonical type 2 cytokines with KH-3 treatment, whereas non–type 2 mediators were relatively preserved. These findings indicate that HuR supports type 2 effector programs in human ILC2s, consistent with a similar pattern observed in human lung Th2 cells (Figure 5).

### Decreased *GATA3* mRNA half-life and altered ILC2 effector-associated transcripts following HuR inhibition (Figure 8)

**Figure 8.**
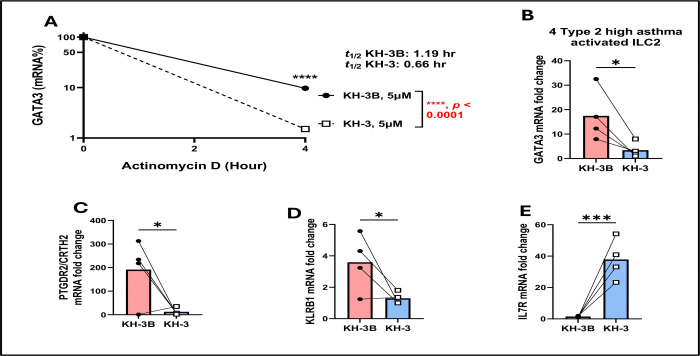
Decreased *GATA3* mRNA half-life and altered ILC2 effector–associated transcripts following HuR inhibition. Actinomycin D (ActD) chase assays, performed following the experimental design described in Figure 7A, demonstrate accelerated decay of *GATA3* mRNA following KH-3 treatment (t½: KH-3B, 1.19 hr; KH-3, 0.66 hr; ****p < 0.0001, two-way ANOVA). **(B–E)** Steady-state mRNA expression of *GATA3*, *PTGDR2*, *KLRB1*, and *IL17R* measured by RT-qPCR. KH-3 significantly reduced *GATA3*, *PTGDR2*, and *KLRB1*, while *IL17R* expression was increased. Data are presented as mean ± SD from independent donors. Statistical comparisons were performed using paired two-tailed Student’s t test. *p < 0.05; ***p < 0.001; ns, not significant.

To determine whether HuR regulates GATA3 post-transcriptionally in human ILC2, we performed ActD chase assays on PBMC-derived ILC2 from type 2–high asthmatic donors treated with KH-3 or KH-3B (Figure 8A). *GATA3* mRNA decayed more rapidly in KH-3–treated ILC2 than in KH-3B controls, with the estimated half-life decreasing from 1.19 hours under KH-3B conditions to 0.66 hours with KH-3 (*p* < 0.0001, two-way ANOVA; Figure 8A), paralleling the KH-3–induced destabilization of GATA3 observed in human lung CD4^+^ T cells (Figure 6B). We next assessed steady-state expression of ILC2-associated genes after HuR inhibition. KH-3 significantly reduced *GATA3* mRNA abundance compared with KH-3B (*p* < 0.05; Figure 8B) and lowered expression of *PTGDR2* (encoding CRTH2) and *KLRB1* (encoding CD161) both *p* < 0.05; Figure 8C, 8D), consistent with a loss of the canonical type 2 ILC2 phenotype. In contrast, *IL7R* (encoding the IL-7 receptor α chain, CD127) showed a significant increase in steady-state mRNA after KH-3 treatment (*p* < 0.001; Figure 8E), suggesting that HuR inhibition may differentially affect cytokine-receptor pathways in ILC2. Together with the Luminex data (Figure 7), these findings indicate that KH-3 constrains ILC2 effector function primarily by destabilizing *GATA3* mRNA and reducing expression of key effector-associated receptors, even though not all ILC2 transcripts exhibit altered mRNA stability.

### Single-cell transcriptomic analysis of BALF links *ELAVL1* and *GATA3* expression to Th2 cells in human allergic airways following allergen challenge (Figure 9)

**Figure 9.**
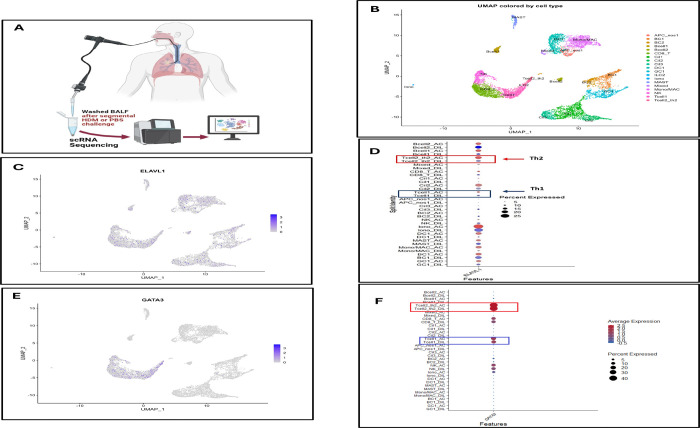
Single-cell transcriptomic analysis of BALF links *ELAVL1* and *GATA3* expression to Th2 cells in human allergic airways following allergen challenge. **(A)** Schematic of experimental workflow. Bronchoalveolar lavage fluid (BALF) was collected following segmental HDM or PBS challenge. Washed BALF cells were processed for single-cell RNA sequencing (scRNA-seq) to characterize airway immune cell populations. **(B)** UMAP visualization of scRNA-seq data colored by annotated immune cell populations, including CD4^+^ T cell subsets, CD8^+^ T cells, B cells, ILC2, dendritic cells, monocytes/macrophages, mast cells, and other immune clusters. Distinct clustering confirms successful resolution of major airway immune populations. **(C)** Feature plot showing expression of *ELAVL1* (encoding HuR) across immune cell populations, demonstrating broad expression with enrichment in T cell clusters. **(D)** Dot plot summarizing *ELAVL1* expression across annotated cell types in HDM-challenged and diluent control samples, showing increased expression in Th2-like CD4^+^ T cell clusters relative to other lymphocyte subsets. **(E)** Feature plot displaying *GATA3* expression across the UMAP projection, localized predominantly to Th2-like CD4^+^ T cell populations. **(F)** Dot plot summarizing *GATA3* expression across immune cell clusters, demonstrating enrichment in Th2 cells and limited expression in non–type 2 immune subsets. Data represent integrated scRNA-seq analysis of BALF immune cells from HDM- and PBS-challenged human airways. Dot size reflects the percentage of cells expressing the gene, and color intensity represents average expression.

To place these mechanistic observations in the context of *in vivo* human airway inflammation, we analyzed single-cell RNA sequencing (scRNA-seq) data from BALF obtained after segmental HDM or control challenge (Figure 9A). Unsupervised clustering and UMAP visualization resolved major immune lineages, including CD4^+^ and CD8^+^ T cell subsets, B cells, ILC2, myeloid cells, mast cells, and other populations (Figure 9B).

*ELAVL1*, the gene encoding HuR, was broadly expressed across immune cells but showed higher expression in T cell–enriched clusters (Figure 9C). Dot plot analysis further confirmed enrichment of *ELAVL1* in Th2-like CD4^+^ T cell clusters relative to other lymphocyte subsets (Figure 9D).

Feature and dot plot analyses of GATA3 demonstrated that its expression was largely confined to Th2-like CD4^+^ T cells, with minimal expression in Th1-like or non–type 2 subsets (Figure 9E, F). The overlap of *ELAVL1* and *GATA3* in these Th2-skewed clusters is consistent with our functional data showing that HuR stabilizes *GATA3* mRNA and supports Th2 effector function in murine and human CD4^+^ T cells (Figures 2, 4, and 5).

While the single-cell data in Figure 9 are associative and do not establish causality, they provide in situ evidence that *ELAVL1* and *GATA3* are co-expressed in CD4^+^ T cell populations that contribute to allergic airway inflammation.

## Discussion

Type 2–high asthma arises from coordinated activation of adaptive Th2 cells and innate ILC2s, yet current biologic therapies predominantly neutralize individual effector cytokines or their receptors rather than upstream mechanisms that restrain both arms of type 2 immunity, and many patients remain symptomatic despite treatment (5–8, 36–38). Here, we identify the RNA-binding protein HuR as an upstream post-transcriptional regulator of this shared type 2 program and show that inhibiting HuR reduces *GATA3* mRNA stability, dampens Th2 and ILC2 cytokine production, and improves allergen-induced airway disease in complementary mouse and human systems (25, 26, 39, 40). These findings support post-transcriptional control of GATA3-dependent circuits as an additional layer of regulation in allergic asthma beyond current cytokine-targeted biologics (6, 26, 36, 39).

Prior studies, including our own, demonstrated that HuR stabilizes *GATA3* transcripts and promotes Th2 cytokine production, mainly using T cell–specific HuR deletion or siRNA in peripheral blood–derived T cells (25, 26, 39, 41). HuR overexpression enhances GATA3 expression and Th2 cytokine production, whereas HuR silencing reduces *GATA3* mRNA stability and attenuates downstream cytokine expression, supporting a central role for HuR in coordinating the Th2 program (25). Here we show that a drug-like small molecule can similarly destabilize murine *Gata3* and human *GATA3* mRNA and suppress downstream Th2 cytokines in both murine and human lung CD4^+^ T cells. This supports a model in which GATA3-driven responses are sustained not only by lineage commitment but also by continued RNA stabilization, with HuR acting as a molecular scaffold that maintains the type 2 transcriptional program in inflamed tissue (19, 22, 25, 26). *In vivo*, KH-3 reduced BALF inflammatory cellularity, eosinophilic airway inflammation, and lung Th2 cytokines, and it improved airway hyperresponsiveness in an HDM-driven model, consistent with prior HuR-loss studies in the T-cell and epithelial compartments of allergen-exposed mice (25, 26, 39–41).

Our human studies demonstrate HuR-dependent regulation of type 2 pathways in primary lung CD4^+^ T cells and circulating ILC2s from asthmatic donors, providing mechanistic insight in disease-relevant lymphocyte populations (26, 38). As direct access to human airway tissue is limited, combining lung-derived CD4^+^ T cells with PBMC-derived ILC2s provides a practical window into HuR-regulated type 2 immunity in humans (38, 42).

Segmental allergen challenge studies have demonstrated dynamic airway responses to allergen exposure and enrichment of type 2 effector populations in BALF from asthmatic patients (43–47). To place our HuR findings in this context, we reanalyzed the single-cell BALF dataset generated by Siddiqui et al. (47), which revealed enrichment of *GATA3*- and *ELAVL1*-associated transcripts in Th2-skewed CD4^+^ T-cell clusters, suggesting activation of type 2–related regulatory programs in the human airway compartment (26, 46). Together with our finding that KH-3 accelerates *GATA3* mRNA decay and reduces Th2 cytokine production in lung-derived CD4^+^ T cells from asthmatic donors, these observations provide evidence that the HuR–GATA3 axis operates in the relevant anatomic compartment in human disease (26). Consistent with classic studies showing that CD4^+^, but not CD8^+^, T cells are the dominant source of Th2-type cytokine mRNA and eosinophil-prolonging factors in blood and BALF of asthmatic patients, we observed broad *ELAVL1* expression with prominent enrichment in GATA3^+^ CD4^+^ T-cell clusters (43, 44, 48). Although these data remain associative, they provide a strong rationale for future perturbation studies combining HuR inhibition with single-cell multiomics to define the HuR-dependent transcriptional network in human airway CD4^+^ T cells, building on existing single-cell maps of allergen-challenged airways (46). These observations further suggest that HuR targeting could complement current biologics and optimized multi-drug inhaler regimens, particularly in patients whose disease remains active despite IL-5, IL-4Rα, or TSLP blockade and single- or triple-inhaler controller therapy (6, 7, 36).

Our results also broaden HuR biology beyond adaptive T cells by implicating it in ILC2 function (26, 40, 49). ILC2s are now recognized as central drivers of type 2 inflammation, especially in exacerbations and disease recurrence, and they cooperate with CD4^+^ T cells to amplify airway pathology (8, 37, 38, 50, 51). Several groups have highlighted their distinct transcriptional and metabolic dependencies, including GATA3- and c-Myc–centered programs and IL-33–driven developmental checkpoints (9–11, 52–57). In particular, an ILC2-restricted tandem super-enhancer is required for stage-specific GATA3 up-regulation and ILC2 maintenance, while sparing Th2 cells (52, 53). We found that KH-3 markedly reduced IL-5, IL-13, IL-9, and related mediators in PBMC-derived ILC2s from asthmatic donors, indicating that HuR supports a shared effector program across Th2 cells and ILC2s rather than a T-cell-restricted pathway. Given accumulating evidence that targeting ILC2s, either directly or via upstream mediators such as TSLP, IL-33, or CRTh2, can mitigate asthma relapse and steroid-refractory disease, HuR inhibition may offer a way to simultaneously restrain both adaptive and innate type 2 compartments (8, 37, 50–52, 54, 55, 58, 59).

An additional strength of HuR inhibition in our system is its preferential effect on type 2–associated programs. Across our models, HuR blockade using KH-3 had modest effects on non–type 2 markers such as *TBX21*, *RORC*, IL-2, or IL-10, consistent with preferential regulation of AU-rich 3′UTR–containing transcripts rather than uniform effects across the transcriptome (19, 21, 22, 25, 26, 49). Similar selectivity has been reported in other disease models, where KH-3 or related HuR inhibitors attenuate pathogenic gene expression in cancer and kidney disease without broad cytotoxicity (27, 30–33, 60). For chronic diseases like asthma, where long-term safety is critical and many patients already receive triple inhaler therapy, the ability to blunt type 2 pathways without broadly paralyzing immune function will be essential (4, 6, 36). Our work also connects pharmacologic HuR inhibition to prior genetic studies. T cell–specific HuR ablation impairs Th2 differentiation and reduces allergen-driven lung inflammation, whereas HuR deficiency in regulatory T cells destabilizes *Foxp3* and compromises suppressive function (26). In the present study, KH-3 recapitulated key features of the Th2-directed HuR knockout, including reduced type 2 cytokine production and improved airway physiology, without directly perturbing Foxp3^+^ Tregs in our experimental systems (26, 49). Together, these observations indicate that HuR regulates CD4^+^ T-cell responses in a context-dependent manner, with a preferential effect on type 2 effector responses in allergic airway inflammation models. Along with reports that HuR controls chemokine production in airway epithelial and smooth muscle cells and contributes to severe asthma–like phenotypes, our findings underscore the breadth of HuR’s influence across structural and immune compartments in the lung and motivate further evaluation of airway-targeted HuR modulation as a potential strategy to dampen pathogenic type 2 responses while carefully monitoring effects on regulatory pathways (26, 36, 40, 49).

Several limitations should be acknowledged. KH-3 was studied in preclinical models and *ex vivo* human cells, and its pharmacokinetics, tissue distribution, and off-target profile *in vivo* remain incompletely defined (27, 30, 31, 60, 61). The compound has physicochemical properties compatible with further optimization, but it is not yet suitable as a clinical candidate; additional medicinal chemistry will be needed to generate analogs with improved potency, metabolic stability, and safety margins (28, 34, 35). Moreover, although we focused on *GATA3* as a mechanistically tractable HuR target, HuR binds hundreds of inflammatory transcripts including mRNAs encoding cytokines, chemokines, receptors, and signaling intermediates, and it is likely that coordinated changes in multiple targets contribute to the overall phenotype (19–22, 25, 26, 40). In contrast to cytokine-directed biologics, such upstream modulation of a shared regulatory hub is expected to have broader effects and may carry a higher risk of off-target or systemic toxicities that require careful definition (5–7, 26, 36, 49). Unbiased transcriptomic and ribosome-profiling approaches after HuR inhibition will be important to map this broader network and to identify pathways that might limit efficacy or cause toxicity (25, 26, 39, 40, 56, 61, 62). Our human cohorts were modest in size and enriched for donors undergoing clinically indicated procedures, which may limit generalizability (42, 46). Larger, prospectively phenotyped cohorts will be needed to determine whether baseline *ELAVL1* or *GATA3* expression, or HuR-dependent gene signatures, identify patients most likely to benefit from HuR-directed therapies (6, 7, 26). In addition, the BALF single-cell analysis is associative; although it supports the presence of HuR-high, GATA3-high Th2 and ILC2 clusters *in vivo*, it does not directly prove that HuR activity is required for their maintenance (46, 56). Perturbational single-cell approaches, such as *ex vivo* HuR inhibition followed by multiome profiling or CRISPR-based perturb-seq, could help move from correlation to causality and may reveal cell-type-specific vulnerabilities (7, 46, 56, 60) These limitations also point toward clear future directions. One priority will be to test KH-3 and next-generation HuR inhibitors in delivery formats that better approximate clinical use, such as inhaled or intranasal administration, which could enrich drug exposure in the airways while limiting systemic effects (6, 36). Parallel efforts should evaluate HuR inhibition in models of viral-induced exacerbation and steroid-refractory asthma, settings where ILC2s and tissue-resident Th2 cells play disproportionate roles and where current biologics and triple inhaler regimens remain imperfect (36, 46, 51). It will also be important to define how HuR intersects with other stress-response and metabolic pathways in Th2 cells and ILC2s, including RBM3, ferroptosis, and mitochondrial fitness, which have recently been implicated in the survival and plasticity of these populations (6, 36, 51, 52, 56, 57, 63)

In summary, our work adds to a growing body of evidence that RNA-binding proteins are critical, yet still underexploited, regulators of immune pathology (19, 20, 56). By destabilizing *GATA3* and additional type 2–associated transcripts, HuR inhibition with KH-3 attenuates Th2 and ILC2 effector function and improves allergen-driven airway disease *in vivo* (25, 26, 39, 40). These findings suggest that targeting post-transcriptional control mechanisms could complement cytokine-blocking biologics and advanced inhaled therapies, offering a broader and potentially more durable strategy for treating type 2–high asthma (6, 7, 26, 36).

## Materials and Methods

### Study design and randomization/blinding

This study was designed to determine whether the RNA-binding protein HuR regulates GATA3-dependent type 2 inflammatory responses characteristic of allergic asthma. To interrogate HuR function, we used the small-molecule HuR inhibitor KH3 as an experimental probe together with its inactive structural analog KH3B as a control. *In vivo* experiments were performed in an HDM-induced airway inflammation mouse model, accompanied by *ex vivo* studies using murine splenic and lung CD4^+^ T cells, human lung CD4^+^ T cells, and human PBMC-derived ILC2s. Single-cell transcriptomic data from bronchoalveolar lavage fluid (BALF) obtained from allergen-challenged asthmatic patients were reanalyzed to evaluate the expression of *ELAVL1* and *GATA3* genes across type 2 inflammatory cell populations in human asthma.

The work comprised four integrated experimental components: (1) HDM mouse model, (2) human lung CD4^+^ T cells, (3) human PBMC-derived ILC2, and (4) BALF scRNA-seq data from asthmatics. All mouse experiments used littermate controls with block randomization by sex and body weight (±2 g). Experimenters were blinded to treatment. No statistical methods were used to predetermine sample size for human studies, which were designed as mechanistic experiments based on prior HuR work. No outliers were excluded; all data are shown. Data collection and analysis were not performed in a completely randomized manner due to biological constraints such as donor availability.

### Sex as a biological variable

Sex as a biological variable. Most *in vivo* experiments were conducted using female mice, consistent with established HDM models of allergic airway inflammation. Pilot studies including both male and female mice did not reveal significant sex-specific differences in the measured outcomes; therefore, subsequent mechanistic experiments were performed primarily in female mice. These data are interpreted with the understanding that key pathways of type 2 inflammation are conserved between sexes. For human studies, samples were obtained from both male and female donors when available; sex was not used as a stratification variable due to sample size and the mechanistic nature of the study.

### Human subjects and ethics

Human donor selection for the lung CD4^+^ T-cell and PBMC studies followed the framework used in our prior HuR asthma study, with subjects classified by asthma status and type 2 inflammatory endotype when applicable. In the earlier work, type 2–high asthma was defined by blood eosinophilia and/or elevated fractional exhaled nitric oxide, and subjects receiving biologic therapy were excluded (26). For the present study, donor samples were obtained from de-identified lung tissue and cryopreserved PBMC specimens under approved institutional protocols with written informed consent. Healthy control and asthmatic donors were processed using matched experimental workflows to minimize technical variation.

Human lung tissue was obtained from Gift of Life under an IRB-exempt de-identified protocol and the University of Michigan. PBMC specimens for ILC2 studies were obtained from a VA Merit Study (CX002491, PI: Atasoy, IRBNet ID: ACORP 1644326). Asthma inclusion criteria followed GINA 2025 type 2-high definitions (blood eosinophils ≥300/μL or FeNO ≥25 ppb, no biologics for ≥6 months, FEV1/FVC < 0.7). Control donors were age- (±5 years), and sex-matched, with FEV1 > 80% predicted. Further details of the inclusion and exclusion criteria for type 2 asthmatic patients and control individuals are provided in Supplementary Table 1. Written informed consent was obtained for all living donors in accordance with 45 CFR 46.116. For single-cell RNA-seq, BALF scRNA-seq data from allergen-challenged asthmatic subjects were re-analyzed as described here; sample collection and human subject oversight, including IRB approval and informed consent, were reported in the work of our collaborators (Siddiqui *et al*) (47).

### Animals and housing

Six- to eight-week-old C57BL/6J mice of both sexes (The Jackson Laboratory; strain #000664) were used. Mice were housed in the University of Michigan Unit for Laboratory Animal Medicine specific-pathogen-free facility under standard laboratory conditions (12-hour light/dark cycle, controlled temperature and humidity) in individually ventilated cages with free access to standard chow and automated LIXIT-supplied drinking water. Sex-balanced cohorts (approximately 50:50 male:female) were used whenever possible. All procedures were performed in accordance with the U.S. National Institutes of Health Guide for the Care and Use of Laboratory Animals and were approved by the University of Michigan ULAM Institutional Animal Care and Use Committee (IACUC protocol PRO00011676).

### HDM-induced allergic airway inflammation model

Allergic airway inflammation was induced using a standardized house dust mite (HDM) extract from Dermatophagoides pteronyssinus (XPB82D3A2.5; Stallergenes Greer, Lenoir, NC, USA; lot 431619). According to the manufacturer, this lyophilized whole-body extract is prepared by bi-level extraction (1:20 and 1:10 w/v in 0.01 M ammonium bicarbonate, dialyzed against distilled water) and supplied as a lyophilized cake containing 20 mg dry weight, 6.05 mg protein, and 55.65 μg Der p 1 per 2.5-mL vial, with an endotoxin content of 20,875 EU/vial. Lyophilized HDM was reconstituted in sterile PBS to a stock concentration of 2.5 mg/mL, aliquoted, and stored at −20 °C until use. Mice were sensitized intraperitoneally on days 0 and 7 with 200 μg HDM in 100 μL sterile PBS; control mice received PBS alone. For allergen challenge, mice were administered 50 μg HDM in 50 μL PBS via the oropharyngeal (intratracheal) route on days 14 and 16 (64). KH-3 (HY-134601, MedChemExpress) was administered during the challenge phase at 100 mg/kg intraperitoneally, a dose selected based on tolerability and previously published safety data. KH-3 was prepared by initial dissolution in 5% ethanol and 5% Tween-80, followed by dilution in PBS. Mice received KH-3 or vehicle every other day from day 8 to day 16. For *in vitro* experiments, KH-3 and the inactive structural analog KH-3B (synthesized by the Xu laboratory) were used. Animals were euthanized 24 hours after the final HDM challenge for collection of bronchoalveolar lavage fluid (BALF) and lung tissue or were assessed for airway function as described below. Lungs designated for histology and imaging were collected following transcardial perfusion with 10% neutral buffered formalin (Epredia).

### Bronchoalveolar lavage and cell counts

Lungs were lavaged with 1 mL PBS containing 0.5 mM EDTA. BALF was pooled and centrifuged at 300 g for 5 min at 4°C. Supernatants were aliquoted and stored at −80°C for cytokine analyses. Cell pellets were resuspended and counted using a Neubauer hemocytometer.

### Lung histology and inflammatory scoring

The left lung or designated lobes were fixed in 10% neutral-buffered formalin, paraffin-embedded, and sectioned at 5 μm. Sections were stained with hematoxylin and eosin to assess peribronchial and perivascular inflammation. Representative images were captured using a Leica DM IRB microscope equipped with an Olympus DP70 digital camera at 20× magnification.

### Cytokine measurement

BALF supernatants, lung homogenates, and activated splenic CD4^+^ T-cell culture supernatants from mice were analyzed using DuoSet ELISA kits (R&D Systems) for IL-4, IL-5, IL-13, and IL-17 according to the manufacturer’s instructions. Cytokine concentrations in lung homogenates were normalized to total protein when appropriate. For human cell culture supernatants, cytokines were quantified using the Human Th9/Th17/Th22 Luminex Performance Assay 18-plex fixed panel (LKTM009B, R&D Systems) according to the manufacturer’s instructions. This magnetic bead–based multiplex assay detects IL-9, CCL20/MIP-3α, CD40 ligand/TNFSF5, GM-CSF, IFN-γ, IL-1β, IL-2, IL-4, IL-5, IL-6, IL-10, IL-12p70, IL-13, IL-15, IL-17A, IL-17E/IL-25, IL-33, and TNF-α.

### Airway physiology

Airway hyperresponsiveness was assessed 24 hours after the final HDM challenge in anesthetized, tracheostomized mice by plethysmography using a Buxco FinePointe resistance/compliance system (Data Sciences International, Wilmington, NC). Total respiratory system resistance (Rn) was recorded in response to increasing doses of nebulized methacholine (0, 10, 20, and 40 mg/mL) delivered via the ventilator, and resistance values (Rn) were analyzed across the methacholine dose–response curve, as described earlier (65).

### Isolation and stimulation of murine CD4^+^ T cells

Splenic CD4^+^ T cells were isolated by magnetic bead separation (Miltenyi Biotec). MACS-purified CD4^+^ T cells were cultured in complete media consisting of RPMI 1640 (Gibco) supplemented with 10% heat-inactivated FBS, 2 mM L-glutamine, 1 mM sodium pyruvate, 55 μM β-mercaptoethanol, and gentamicin. Cells were pretreated with KH-3 or inactive analogue KH-3B (2 μM, for 2 hours) and then stimulated with plate-bound anti-CD3 plus soluble anti-CD28 (both from Invitrogen) for up to 4 days. For short-term assays, cells were harvested after 4 days of stimulation for intracellular cytokine staining by flow cytometry and for measurement of secreted cytokines in culture supernatants by ELISA (26).

### Human lung CD4^+^ T-cell isolation and activation

Fresh de-identified human lung tissue (University of Michigan Lung Biorepository or GIFT of Life) was mechanically and enzymatically dissociated to obtain single-cell suspensions, digested in collagenase A and DNase I at 37°C, processed on a gentleMACS Dissociator, and filtered through 70 μm strainers. CD4^+^ T cells were enriched using Human CD4 MicroBeads on LS columns (Miltenyi) in MACS buffer, achieving >95% purity by CD3/CD4 flow cytometry. Purified CD4^+^ T cells were cultured in complete media, pretreated for 2 hours with KH-3 or KH-3B, and then stimulated for 4 days on plates coated with anti-CD3 (5 μg/mL) and anti-CD28 (2 μg/mL). Supernatants were collected for multiplex cytokine assays, and cell pellets were used for RNA extraction and qPCR; in selected experiments, actinomycin D; ActD (5 μg/mL) was added after 4 days of activation to assess mRNA decay.

### Human PBMC-derived ILC2 isolation, culture, and stimulation

Cryopreserved PBMCs from healthy controls and type 2–high asthmatic donors were thawed and recovered in parallel under identical conditions. PBMCs were originally isolated from EDTA-anticoagulated blood by density-gradient centrifugation over Ficoll-Paque PLUS (17–1440-03, GE Healthcare; 16 mL blood layered over 12 mL Ficoll, centrifuged at 2000 ×g for 40 min at room temperature with no brake). After thawing, PBMCs were rested overnight in complete medium supplemented with 10 ng/mL IL-7.

ILC2s were isolated using the Human ILC2 Isolation Kit (Miltenyi Biotec, 130–114-825) according to the manufacturer’s instructions. Peripheral blood mononuclear cells were first incubated with a biotin-conjugated lineage antibody cocktail containing antibodies against CD2, CD3, CD14, CD16, CD19, CD56, CD235a (Glycophorin A), and CD123, followed by Anti-Biotin MicroBeads, and lineage-positive cells were depleted over LS MACS columns. The resulting lineage-negative fraction was then stained with CD294 (CRTH2)-PE and labeled with Anti-PE MicroBeads, and CD294^+^ cells were positively selected on MS MACS columns to enrich for ILC2s. This two-step procedure routinely yielded lineage-negative, CD294^+^ cells at approximately 3 × 10^3^ cells per 50 mL blood. For flow cytometric characterization, enriched cells were further confirmed to be Lin⁻CD45^+^CD127^+^CD161^+^CD294^+^.

Purified ILC2s were cultured in complete medium, pretreated with KH-3 or the inactive analog KH-3B for 2 hours, and then stimulated with recombinant human IL-7, IL-33, and TSLP (10 ng/mL each) for 4 days without compound washout. Cell-free supernatants were collected and stored at −80 °C for multiplex cytokine (Luminex) analysis, and cells were harvested for RNA extraction and qPCR.

On day 4, parallel cultures were subjected to ActD mRNA decay assays. At time 0 (immediately before ActD addition), supernatants were collected for Luminex analysis. Cultures were then treated with ActD and incubated for 4 hours, followed by collection of supernatants. Cells were lysed directly in RLT buffer and stored at −80 °C until RNA isolation and downstream qPCR analysis.

### RNA extraction, qPCR, primer sequences, and mRNA stability

Total RNA from murine and human cells (including CD4^+^ T cells and ILC2s) was isolated using RNeasy Mini kits (Qiagen) according to the manufacturer’s instructions. RNA integrity was verified by spectrophotometric analysis (NanoDrop), and only samples with acceptable quality metrics (A260/A280 ratios of 1.8–2.1 and A260/A230 ratios ≥1.8) were used for downstream analyses.

For standard expression analysis, cDNA was synthesized using the High-Capacity cDNA Reverse Transcription Kit (Applied Biosystems, catalog 4368814) with 10 ng–1 μg RNA per reaction, depending on sample type. Quantitative PCR was performed on a QuantStudio 3 Real-Time PCR System (Applied Biosystems, Foster City, CA) using SYBR Green chemistry with validated primer pairs (Integrated DNA Technologies; HPLC-purified, 500 nM final primer concentration). Relative mRNA expression was calculated using the ΔΔCt method, with *Gapdh* (mouse) or *GAPDH* (human) serving as the internal reference gene.

For mRNA stability assays, mouse and human cells were treated with ActD (5 μg/mL) to block transcription after the indicated activation period, following our previously published protocol (49). RNA was harvested at serial time points after ActD addition (0–4 hours), converted to cDNA, and target transcript abundance was quantified by qPCR as described above. For each transcript, values at time zero were set to 100%, and remaining mRNA at subsequent time points was expressed relative to this baseline; decay curves were fitted using nonlinear regression, and mRNA half-life (t₁/₂) was calculated from the best-fit model.

Primer sequences and amplicon sizes for all mouse and human targets are listed in Tables 1 and 2.

### Flow cytometry

CD4^+^ T cells were isolated from spleens of C57BL/6 mice and activated *in vitro* for 4 days with plate-bound anti-CD3 and soluble anti-CD28 before processing for flow cytometry. Prior to staining, cells were incubated with TruStain FcX Fc receptor–blocking solution (BioLegend) to minimize nonspecific binding and then labeled with LIVE/DEAD^™^ Fixable Dead Cell Stain (Invitrogen) compatible with the multicolor panel. Surface staining was performed at 4°C for 30 minutes in the dark using fluorochrome-conjugated antibodies against CD3 and CD4. For intracellular staining of cytokines and GATA3, cells were fixed and permeabilized using an intracellular staining buffer set (eBioscience), followed by staining with fluorochrome-conjugated anti-cytokine and anti-GATA3 antibodies. Fluorescence-minus-one, single-color, and isotype controls were included for gate setting and compensation, and UltraComp eBeads (Invitrogen) were used for compensation. Data were acquired on a BD FACSCanto flow cytometer and analyzed using FlowJo software (Tree Star) (49).

### Single-cell RNA sequencing

BALF cells obtained after segmental allergen challenge were processed for single-cell RNA sequencing using a 10x Genomics Chromium platform and Illumina sequencing, following the methods described by Siddiqui et al (47), with minor modifications. Briefly, raw data were processed with CellRanger using the GRCh38 reference genome, and downstream analyses were performed in Seurat, including normalization, dimensionality reduction, clustering, and differential expression testing with false-discovery-rate correction; *ELAVL1* (HuR) and *GATA3* expression were then examined across immune cell clusters to assess enrichment in Th2-associated populations.

### Statistical analysis

Unless otherwise specified, data are presented as mean ± SD, as indicated in figure legends. Comparisons between two groups were performed using unpaired or paired Student’s t tests. Multiple-group comparisons were analyzed by one-way or two-way ANOVA with appropriate post hoc tests, as indicated in figure legends. For scRNA-seq, differential expression was assessed using Wilcoxon rank-sum tests with FDR correction (FDR < 0.05) (47). Exact p values are reported when feasible; statistical significance was defined as *p* < 0.05.

## Supplementary Material

Supplement 1

## Figures and Tables

**Table 1.** Mouse qPCR primer sequences and amplicon sizes.

Target: Mouse	Forward primer (5’–3’)	Reverse primer (5’–3’)	Amplicon Size (bp)
*Il4*	CCACGGATGCGACAAAAATCA	TTTGGCACATCCATCTCCGT	96
*Il13*	TGTGTCTCTCCCTCTGACCC	GCCAGGTCCACACTCCATAC	108
*Gata3*	GAGGAGGAACGCTAATGGGG	CGGGTCTGGATGCCTTCTTT	100/108*
*Rorc*	GTGGAGTTTGCCAAGCGGCTTT	CCTGCACATTCTGACTAGGACG	108
*Tbx21*	GGTTGGAGGTGTCTGGGAAG	TAATGGCTTGTGGGCTCCAG	160
*Foxp3*	CTCGCATGTTCGCCTACTTC	CTCTCCACTCGCACAAAGCA	97
*Il17a*	CAGACTACCTCAACCGTTCCAC	TCCAGCTTTCCCTCCGCATTGA	130
*Ifnγ*	CAGCAACAGCAAGGCGAAAA	ATCTCTTCCCCACCCCGAAT	179
*Il10*	CGGGAAGACAATAACTGCACCC	CGGTTAGCAGTATGTTGTCCAGC	130
*Gapdh*	CATCACTGCCACCCAGAAGACTG	ATGCCAGTGAGCTTCCCGTTCAG	153

* Primers amplify multiple Gata3 transcript variants (predicted 100 and 108 bp) with a single specific melt-curve peak.

**Table 2.** Human qPCR primer sequences and amplicon sizes.

Target: Human	Forward primer (5’–3’)	Reverse primer (5’–3’)	Amplicon Size (bp)
*GATA3*	ACCACAACCACACTCTGGAGGA	TCGGTTTCTGGTCTGGATGCCT	124/132*
*RORC*	GCTGAGAAGGACAGGGAGC	GACGACTTGTCCCCACAGATT	99
*TBX21*	CCACCTGTTGTGGTCCAAGT	CATCCTGTAGTGGCTGGTGG	127
*PTGDR2*	TGGAGTCATCCTCTTCGTGGTG	AGTAGGTGAAGAAGGGCAGGGA	119
*KLRB1*	GTTCCACCAAAGAATCCAGCCTG	AAGAGCCGTTTATCCACTTCCAG	150
*IL7R*	ATCGCAGCACTCACTGACCTGT	TCAGGCACTTTACCTCCACGAG	101
*GAPDH*	CCCACTCCTCCACCTTTGAC	TCCTCTTGTGCTCTTGCTGG	179

* Primers amplify multiple GATA3 transcript variants (predicted 124 and 132 bp) with a single specific melt-curve peak.

## Data Availability

All data generated or analyzed during this study are included in this article and its supplementary information files. Raw datasets are available from the corresponding author upon request.

## References

[1] Global Initiative for Asthma (GINA). Global Strategy for Asthma Management and Prevention. 2025 update. Available from: https://ginasthma.org.

[2] Rijavec M, Krumpestar T, Skrgat S, Kern I, Korosec P. T2-high Asthma, Classified by Sputum mRNA Expression of IL4, IL5, and IL13, is Characterized by Eosinophilia and Severe Phenotype. Life (Basel). 2021;11(2). 10.3390/life11020092 PMC7911289 33513844

[3] Pelaia C, Heffler E, Crimi C, Maglio A, Vatrella A, Pelaia G, Interleukins 4 and 13 in Asthma: Key Pathophysiologic Cytokines and Druggable Molecular Targets. Front Pharmacol. 2022;13:851940. 35350765 10.3389/fphar.2022.851940 PMC8957960

[4] Bignold RE, Busby H, Holloway J, Kasu A, Sian S, Johnson JR. Biologic therapies targeting type 2 cytokines are effective at improving asthma symptoms and control-a systematic review and meta-analysis. J Allergy Clin Immunol Glob. 2025;4(1):100374. 39844912 10.1016/j.jacig.2024.100374 PMC11751513

[5] Fahy JV. Type 2 inflammation in asthma--present in most, absent in many. Nat Rev Immunol. 2015;15(1):57–65. 25534623 10.1038/nri3786 PMC4390063

[6] Peters MC, Wenzel SE. Intersection of biology and therapeutics: type 2 targeted therapeutics for adult asthma. Lancet. 2020;395(10221):371–83. 32007172 10.1016/S0140-6736(19)33005-3 PMC8522504

[7] Sim S, Choi Y, Park HS. Immunologic Basis of Type 2 Biologics for Severe Asthma. Immune Netw. 2022;22(6):e45. 36627938 10.4110/in.2022.22.e45 PMC9807964

[8] Rodriguez-Rodriguez N, Gogoi M, McKenzie ANJ. Group 2 Innate Lymphoid Cells: Team Players in Regulating Asthma. Annu Rev Immunol. 2021;39:167–98. 33534604 10.1146/annurev-immunol-110119-091711 PMC7614118

[9] Tindemans I, Serafini N, Di Santo JP, Hendriks RW. GATA-3 function in innate and adaptive immunity. Immunity. 2014;41(2):191–206. 25148023 10.1016/j.immuni.2014.06.006

[10] Zhu J. GATA3 Regulates the Development and Functions of Innate Lymphoid Cell Subsets at Multiple Stages. Front Immunol. 2017;8:1571. 29184556 10.3389/fimmu.2017.01571 PMC5694433

[11] Wan YY. GATA3: a master of many trades in immune regulation. Trends Immunol. 2014;35(6):233–42. 24786134 10.1016/j.it.2014.04.002 PMC4045638

[12] Zheng W, Flavell RA. The transcription factor GATA-3 is necessary and sufficient for Th2 cytokine gene expression in CD4 T cells. Cell. 1997;89(4):587–96. 9160750 10.1016/s0092-8674(00)80240-8

[13] Hoyler T, Klose CS, Souabni A, Turqueti-Neves A, Pfeifer D, Rawlins EL, The transcription factor GATA-3 controls cell fate and maintenance of type 2 innate lymphoid cells. Immunity. 2012;37(4):634–48. 23063333 10.1016/j.immuni.2012.06.020 PMC3662874

[14] Eberl G, Di Santo JP, Vivier E. The brave new world of innate lymphoid cells. Nat Immunol. 2015;16(1):1–5. 25521670 10.1038/ni.3059

[15] Meininger I, Carrasco A, Rao A, Soini T, Kokkinou E, Mjosberg J. Tissue-Specific Features of Innate Lymphoid Cells. Trends Immunol. 2020;41(10):902–17. 32917510 10.1016/j.it.2020.08.009

[16] Moro K, Yamada T, Tanabe M, Takeuchi T, Ikawa T, Kawamoto H, Innate production of T(H)2 cytokines by adipose tissue-associated c-Kit(+)Sca-1(+) lymphoid cells. Nature. 2010;463(7280):540–4. 20023630 10.1038/nature08636

[17] Halim TY, Steer CA, Matha L, Gold MJ, Martinez-Gonzalez I, McNagny KM, Group 2 innate lymphoid cells are critical for the initiation of adaptive T helper 2 cell-mediated allergic lung inflammation. Immunity. 2014;40(3):425–35. 24613091 10.1016/j.immuni.2014.01.011 PMC4210641

[18] Yagi R, Zhong C, Northrup DL, Yu F, Bouladoux N, Spencer S, The transcription factor GATA3 is critical for the development of all IL-7Ralpha-expressing innate lymphoid cells. Immunity. 2014;40(3):378–88. 24631153 10.1016/j.immuni.2014.01.012 PMC4026797

[19] Keene JD. RNA regulons: coordination of post-transcriptional events. Nat Rev Genet. 2007;8(7):533–43. 17572691 10.1038/nrg2111

[20] Fu M, Blackshear PJ. RNA-binding proteins in immune regulation: a focus on CCCH zinc finger proteins. Nat Rev Immunol. 2017;17(2):130–43. 27990022 10.1038/nri.2016.129 PMC5556700

[21] Peng SS, Chen CY, Xu N, Shyu AB. RNA stabilization by the AU-rich element binding protein, HuR, an ELAV protein. EMBO J. 1998;17(12):3461–70. 9628881 10.1093/emboj/17.12.3461 PMC1170682

[22] Simone LE, Keene JD. Mechanisms coordinating ELAV/Hu mRNA regulons. Curr Opin Genet Dev. 2013;23(1):35–43. 23312841 10.1016/j.gde.2012.12.006 PMC3617084

[23] Atasoy U, Watson J, Patel D, Keene JD. ELAV protein HuA (HuR) can redistribute between nucleus and cytoplasm and is upregulated during serum stimulation and T cell activation. J Cell Sci. 1998;111 ( Pt 21):3145–56. 9763509 10.1242/jcs.111.21.3145

[24] Ma WJ, Cheng S, Campbell C, Wright A, Furneaux H. Cloning and characterization of HuR, a ubiquitously expressed Elav-like protein. J Biol Chem. 1996;271(14):8144–51. 8626503 10.1074/jbc.271.14.8144

[25] Stellato C, Gubin MM, Magee JD, Fang X, Fan J, Tartar DM, Coordinate regulation of GATA-3 and Th2 cytokine gene expression by the RNA-binding protein HuR. J Immunol. 2011;187(1):441–9. 21613615 10.4049/jimmunol.1001881 PMC5801757

[26] Fattahi F, Ellis JS, Sylvester M, Bahleda K, Hietanen S, Correa L, HuR-Targeted Inhibition Impairs Th2 Proinflammatory Responses in Asthmatic CD4(+) T Cells. J Immunol. 2022;208(1):38–48. 34862257 10.4049/jimmunol.2100635

[27] Green LC, Anthony SR, Slone S, Lanzillotta L, Nieman ML, Wu X, Human antigen R as a therapeutic target in pathological cardiac hypertrophy. JCI Insight. 2019;4(4). 10.1172/jci.insight.121541 PMC6478406 30668549

[28] Rzeszutek I, Nowak-Krol A. HuR-Targeted Small-Molecule Inhibitors horizontal line Beneficial Impact in Cancer Therapy. J Med Chem. 2025;68(21):22009–32. 41105730 10.1021/acs.jmedchem.5c01487

[29] Guichard V, Leao FB, Zhao J, Zhang Y, Ito T, Shirley S, Pre-existing epigenetic state and differential NF-kappaB activation shape type 2 immune cell responses. Immunity. 2025;58(6):1422–37 e6. 40367949 10.1016/j.immuni.2025.04.016 PMC12626339

[30] Dong R, Chen P, Polireddy K, Wu X, Wang T, Ramesh R, An RNA-Binding Protein, Hu-antigen R, in Pancreatic Cancer Epithelial to Mesenchymal Transition, Metastasis, and Cancer Stem Cells. Mol Cancer Ther. 2020;19(11):2267–77. 32879054 10.1158/1535-7163.MCT-19-0822 PMC7921213

[31] Arneson-Wissink PC, Pelz K, Worley B, Mendez H, Pham P, Diba P, Impaired Adipose Anabolism in Pancreatic Cancer Cachexia Is Reversed by HuR Inhibition. J Cachexia Sarcopenia Muscle. 2026;17(2):e70253. 41852200 10.1002/jcsm.70253 PMC13140750

[32] Liu S, Huang Z, Tang A, Wu X, Aube J, Xu L, Inhibition of RNA-binding protein HuR reduces glomerulosclerosis in experimental nephritis. Clin Sci (Lond). 2020;134(12):1433–48. 32478392 10.1042/CS20200193 PMC8086301

[33] Wu X, Gardashova G, Lan L, Han S, Zhong C, Marquez RT, Targeting the interaction between RNA-binding protein HuR and FOXQ1 suppresses breast cancer invasion and metastasis. Commun Biol. 2020;3(1):193. 32332873 10.1038/s42003-020-0933-1 PMC7181695

[34] Muralidharan R, Mehta M, Ahmed R, Roy S, Xu L, Aube J, HuR-targeted small molecule inhibitor exhibits cytotoxicity towards human lung cancer cells. Sci Rep. 2017;7(1):9694. 28855578 10.1038/s41598-017-07787-4 PMC5577245

[35] Wu X, Lan L, Wilson DM, Marquez RT, Tsao WC, Gao P, Identification and validation of novel small molecule disruptors of HuR-mRNA interaction. ACS Chem Biol. 2015;10(6):1476–84. 25750985 10.1021/cb500851u PMC4631057

[36] Agusti A, Fabbri L, Lahousse L, Singh D, Papi A. Single inhaler triple therapy (SITT) in asthma: Systematic review and practice implications. Allergy. 2022;77(4):1105–13. 34478578 10.1111/all.15076 PMC9290056

[37] van Rijt L, von Richthofen H, van Ree R. Type 2 innate lymphoid cells: at the cross-roads in allergic asthma. Semin Immunopathol. 2016;38(4):483–96. 26965110 10.1007/s00281-016-0556-2 PMC4896999

[38] Verma M, Verma D, Alam R. Role of type-2 innate lymphoid cells (ILC2s) in type-2 asthma. Curr Opin Allergy Clin Immunol. 2022;22(1):29–35. 34840275 10.1097/ACI.0000000000000798 PMC8926081

[39] Licata LA, Hostetter CL, Crismale J, Sheth A, Keen JC. The RNA-binding protein HuR regulates GATA3 mRNA stability in human breast cancer cell lines. Breast Cancer Res Treat. 2010;122(1):55–63. 19728080 10.1007/s10549-009-0517-8

[40] Herjan T, Xiao J, Dziendziel Kolanek M. RNA-Binding Protein HuR Promotes Airway Inflammation in a House Dust Mite-Induced Allergic Asthma Model. J Interferon Cytokine Res. 2022;42(1):29–38. 35041516 10.1089/jir.2021.0171 PMC8787712

[41] Gubin MM, Techasintana P, Magee JD, Dahm GM, Calaluce R, Martindale JL, Conditional knockout of the RNA-binding protein HuR in CD4(+) T cells reveals a gene dosage effect on cytokine production. Mol Med. 2014;20(1):93–108. 24477678 10.2119/molmed.2013.00127 PMC3960399

[42] Edwards J, Belvisi M, Dahlen SE, Holgate S, Holmes A. Human tissue models for a human disease: what are the barriers? Thorax. 2015;70(7):695–7. 25631322 10.1136/thoraxjnl-2014-206648 PMC4483787

[43] Robinson D, Hamid Q, Bentley A, Ying S, Kay AB, Durham SR. Activation of CD4+ T cells, increased TH2-type cytokine mRNA expression, and eosinophil recruitment in bronchoalveolar lavage after allergen inhalation challenge in patients with atopic asthma. J Allergy Clin Immunol. 1993;92(2):313–24. 8349942 10.1016/0091-6749(93)90175-f

[44] Krug N, Erpenbeck VJ, Balke K, Petschallies J, Tschernig T, Hohlfeld JM, Cytokine profile of bronchoalveolar lavage-derived CD4(+), CD8(+), and gammadelta T cells in people with asthma after segmental allergen challenge. Am J Respir Cell Mol Biol. 2001;25(1):125–31. 11472985 10.1165/ajrcmb.25.1.4194

[45] Cho JL, Ling MF, Adams DC, Faustino L, Islam SA, Afshar R, Allergic asthma is distinguished by sensitivity of allergen-specific CD4+ T cells and airway structural cells to type 2 inflammation. Sci Transl Med. 2016;8(359):359ra132. 10.1126/scitranslmed.aag1370 PMC5399547 27708065

[46] Alladina J, Smith NP, Kooistra T, Slowikowski K, Kernin IJ, Deguine J, A human model of asthma exacerbation reveals transcriptional programs and cell circuits specific to allergic asthma. Sci Immunol. 2023;8(83):eabq6352. 37146132 10.1126/sciimmunol.abq6352 PMC10440046

[47] Siddiqui S, Johansson K, Joo A, Bonser LR, Koh KD, Le Tonqueze O, Epithelial miR-141 regulates IL-13-induced airway mucus production. JCI Insight. 2021;6(5). 10.1172/jci.insight.139019 PMC8021117 33682796

[48] Corrigan CJ, Hamid Q, North J, Barkans J, Moqbel R, Durham S, Peripheral blood CD4 but not CD8 t-lymphocytes in patients with exacerbation of asthma transcribe and translate messenger RNA encoding cytokines which prolong eosinophil survival in the context of a Th2-type pattern: effect of glucocorticoid therapy. Am J Respir Cell Mol Biol. 1995;12(5):567–78. 7742019 10.1165/ajrcmb.12.5.7742019

[49] Fattahi F, Ellis JS, Vallance L, Bahleda K, Holden J, Socha S, HuR ablation destabilizes Foxp3 mRNA and impairs regulatory T cell function, contributing to an autoimmune phenotype. Front Immunol. 2025;16:1618677. 41080546 10.3389/fimmu.2025.1618677 PMC12511036

[50] Drake LY, Iijima K, Kita H. Group 2 innate lymphoid cells and CD4+ T cells cooperate to mediate type 2 immune response in mice. Allergy. 2014;69(10):1300–7. 24939388 10.1111/all.12446 PMC4160406

[51] Fonseca W, Lukacs NW, Elesela S, Malinczak CA. Role of ILC2 in Viral-Induced Lung Pathogenesis. Front Immunol. 2021;12:675169. 33953732 10.3389/fimmu.2021.675169 PMC8092393

[52] Steer CA, Matha L, Shim H, Takei F. Lung group 2 innate lymphoid cells are trained by endogenous IL-33 in the neonatal period. JCI Insight. 2020;5(14). 10.1172/jci.insight.135961 PMC7453888 32573494

[53] Furuya H, Toda Y, Iwata A, Kanai M, Kato K, Kumagai T, Stage-specific GATA3 induction promotes ILC2 development after lineage commitment. Nat Commun. 2024;15(1):5610. 38969652 10.1038/s41467-024-49881-y PMC11226602

[54] Ye L, Pan J, Liang M, Pasha MA, Shen X, D’Souza SS, A critical role for c-Myc in group 2 innate lymphoid cell activation. Allergy. 2020;75(4):841–52. 31833571 10.1111/all.14149 PMC7176544

[55] Thio CL, Chang YJ. The modulation of pulmonary group 2 innate lymphoid cell function in asthma: from inflammatory mediators to environmental and metabolic factors. Exp Mol Med. 2023;55(9):1872–84. 37696890 10.1038/s12276-023-01021-0 PMC10545775

[56] Badrani JH, Strohm AN, Lacasa L, Civello B, Cavagnero K, Haung YA, RNA-binding protein RBM3 intrinsically suppresses lung innate lymphoid cell activation and inflammation partially through CysLT1R. Nat Commun. 2022;13(1):4435. 35908044 10.1038/s41467-022-32176-5 PMC9338970

[57] Wientjens C, Doverman M, Zurkovic J, More T, Surendar J, Nesic S, Tolerance to ferroptosis facilitates lipid metabolism and pathogenic type 2 immunity in allergic airway inflammation. Immunity. 2026;59(2):322–38 e9. 41380684 10.1016/j.immuni.2025.11.018

[58] Aron JL, Akbari O. Regulatory T cells and type 2 innate lymphoid cell-dependent asthma. Allergy. 2017;72(8):1148–55. 28160290 10.1111/all.13139

[59] Huang T, Hazen M, Shang Y, Zhou M, Wu X, Yan D, Depletion of major pathogenic cells in asthma by targeting CRTh2. JCI Insight. 2016;1(7):e86689. 27699264 10.1172/jci.insight.86689 PMC5033936

[60] Wei L, Kim SH, Armaly AM, Aube J, Xu L, Wu X. RNA-binding protein HuR inhibition induces multiple programmed cell death in breast and prostate cancer. Cell Commun Signal. 2024;22(1):580. 39627778 10.1186/s12964-024-01916-z PMC11613925

[61] Liu GY, Sabatini DM. mTOR at the nexus of nutrition, growth, ageing and disease. Nat Rev Mol Cell Biol. 2020;21(4):183–203. 31937935 10.1038/s41580-019-0199-y PMC7102936

[62] Techasintana P, Davis JW, Gubin MM, Magee JD, Atasoy U. Transcriptomic-Wide Discovery of Direct and Indirect HuR RNA Targets in Activated CD4+ T Cells. PLoS One. 2015;10(7):e0129321. 26162078 10.1371/journal.pone.0129321 PMC4498740

[63] Lv X, Tang W, Qin J, Wang W, Dong J, Wei Y. The crosslinks between ferroptosis and autophagy in asthma. Front Immunol. 2023;14:1140791. 37063888 10.3389/fimmu.2023.1140791 PMC10090423

[64] Akkenepally SV, Yombo DJK, Yerubandi S, Reddy GB, Deshpande DA, McCormack FX, Interleukin 31 receptor alpha promotes smooth muscle cell contraction and airway hyperresponsiveness in asthma. Nat Commun. 2023;14(1):8207. 38081868 10.1038/s41467-023-44040-1 PMC10713652

[65] Schneider D, Hong JY, Popova AP, Bowman ER, Linn MJ, McLean AM, Neonatal rhinovirus infection induces mucous metaplasia and airways hyperresponsiveness. J Immunol. 2012;188(6):2894–904. 22331068 10.4049/jimmunol.1101391 PMC3294163

