# Supplementary material for: HuR Regulates GATA3-Driven Type 2 Inflammation in CD4+ T cells and ILC2 in Airway Inflammation
Source: bioRxiv. 2026 May 14:2026.04.23.720195. Originally published 2026 Apr 27. Preprint. [Version 2] doi: 10.64898/2026.04.23.720195 (PMC13142492; doi:10.64898/2026.04.23.720195)

Graphical Abstract

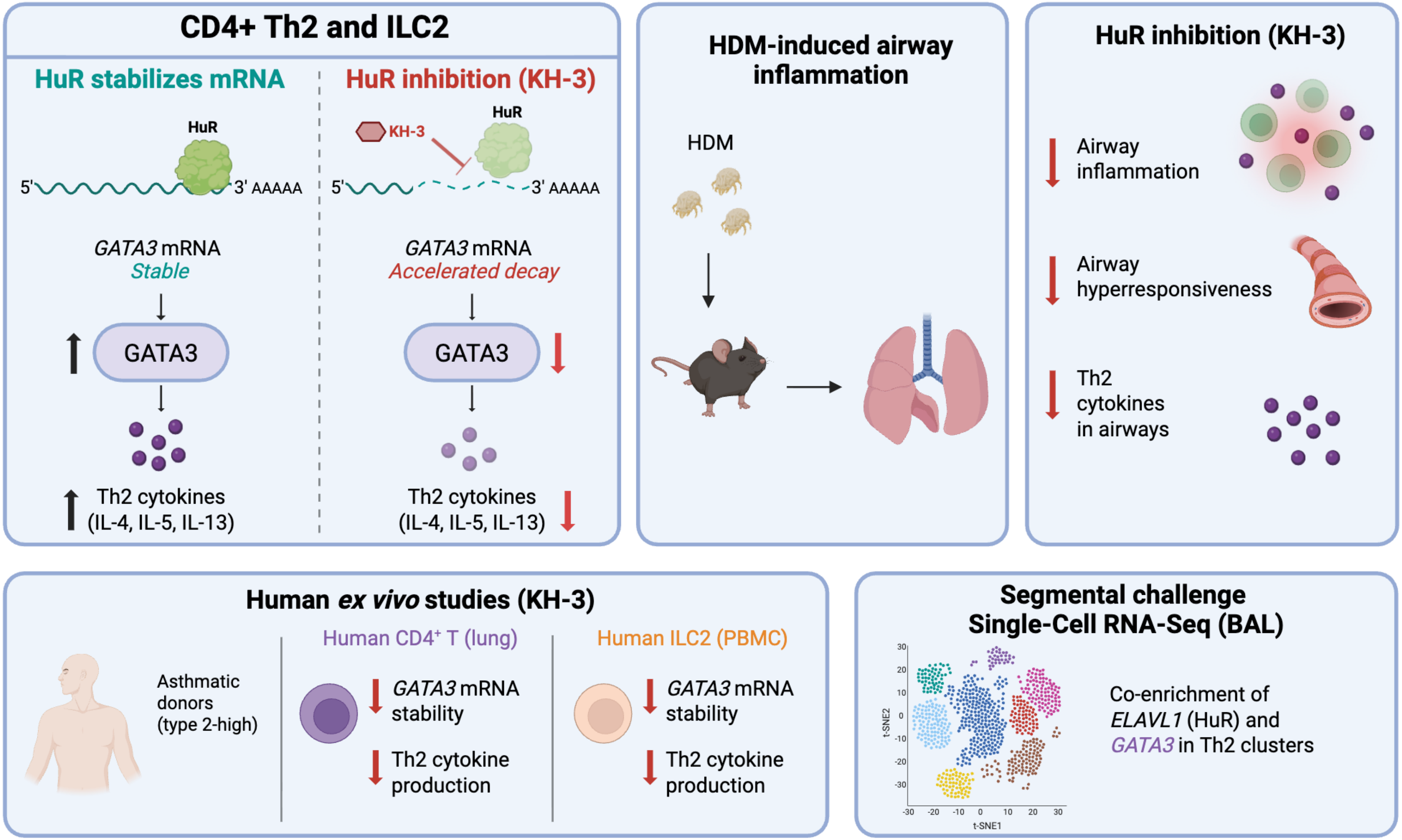

Supplement: Supplement 1 [file media-1.pdf]
